# The Genetic Architecture of Noise-Induced Hearing Loss: Evidence for a Gene-by-Environment Interaction

**DOI:** 10.1534/g3.116.032516

**Published:** 2016-08-11

**Authors:** Joel Lavinsky, Marshall Ge, Amanda L. Crow, Calvin Pan, Juemei Wang, Pezhman Salehi, Anthony Myint, Eleazar Eskin, Hooman Allayee, Aldons J. Lusis, Rick A. Friedman

**Affiliations:** * Tina and Rick Caruso Department of Otolaryngology, Zilkha Neurogenetic Institute; ‡ Department of Preventive Medicine and Institute for Genetic Medicine, USC Keck School of Medicine, University of Southern California, Los Angeles, California 90033; † Graduate Program in Surgical Sciences, Federal University of Rio Grande do Sul, Porto Alegre, Rio Grande do Sul, Brazil; § Department of Human Genetics; ** Department of Computer Science; †† Department of Microbiology, Immunology, and Molecular Genetics, University of California, Los Angeles, California 90024

**Keywords:** GWAS, NIHL, gene-by-environment, HMDP, eQTL

## Abstract

The discovery of environmentally specific genetic effects is crucial to the understanding of complex traits, such as susceptibility to noise-induced hearing loss (NIHL). We describe the first genome-wide association study (GWAS) for NIHL in a large and well-characterized population of inbred mouse strains, known as the Hybrid Mouse Diversity Panel (HMDP). We recorded auditory brainstem response (ABR) thresholds both pre and post 2-hr exposure to 10-kHz octave band noise at 108 dB sound pressure level in 5–6-wk-old female mice from the HMDP (4–5 mice/strain). From the observation that NIHL susceptibility varied among the strains, we performed a GWAS with correction for population structure and mapped a locus on chromosome 6 that was statistically significantly associated with two adjacent frequencies. We then used a “genetical genomics” approach that included the analysis of cochlear eQTLs to identify candidate genes within the GWAS QTL. In order to validate the gene-by-environment interaction, we compared the effects of the postnoise exposure locus with that from the same unexposed strains. The most significant SNP at chromosome 6 (rs37517079) was associated with noise susceptibility, but was not significant at the same frequencies in our unexposed study. These findings demonstrate that the genetic architecture of NIHL is distinct from that of unexposed hearing levels and provide strong evidence for gene-by-environment interactions in NIHL.

Noise-induced hearing loss (NIHL) is the most common occupational disease in the world and the second leading cause of sensorineural hearing loss (Conway *et al.* 1993). It has been estimated that worldwide, as many as 500 million individuals might be at risk of hearing loss from noise exposure (Sliwinska-Kowalska and Pawelczyk 2013). Susceptibility to NIHL differs among individuals and appears to be due to a combination of genetic and environmental factors. We have recently reported a comprehensive analysis of 100 strains of noise-exposed and unexposed mice and, utilizing these data, we have described the genetic architecture of baseline hearing loss in these same strains (Crow *et al.*, 2015; Myint *et al.*, 2016).

The elucidation of environmentally specific genetic effects is critical to the understanding of complex traits such as NIHL. In humans, gene-by-environment (G × E) interactions are being considered (Kang *et al.* 2014), but few of the studies have been replicated to date. A major hurdle to human studies is the inability to control and quantitate environmental exposures in a consistent fashion, which leads to the lack of well-characterized and monitored populations for study. This is notably the case for NIHL, and only a few modestly characterized and underpowered studies have been published (Sliwinska-Kowalska and Pawelczyk 2013). It is for this reason that many investigators are turning toward model organisms, such as mice, allowing them to circumvent these limitations.

Mouse models have several advantages over human studies: the environment can be more carefully controlled, measurements can be replicated in genetically identical animals, the environmental effect on traits can be reduced, and the proportion of the variability explained by genetic variation is increased. Complex traits in mouse strains have been shown to have higher heritability, and genetic loci often have stronger effects compared to humans (Lindblad-Toh *et al.* 2000; Yalcin *et al.* 2004).

We have employed a genome-wide association study (GWAS) with correction for population structure using the Hybrid Mouse Diversity Panel (HMDP) to study genetic variation in hearing phenotypes (Lavinsky *et al.* 2015; Crow *et al.* 2015). The HMDP is a collection of common inbred strains providing sufficient power to map loci responsible for as little as 5% of the phenotypic variance (Flint and Eskin 2012; Ghazalpour *et al.* 2012). This resource is composed of 30 classical inbred strains (CI) providing genetic resolution and allelic diversity, and 70 recombinant inbred strains (RI) that enhance resolution and dramatically increase power (Ghazalpour *et al.* 2012; Rau *et al.* 2015). Association was carried out after correcting for population structure using efficient mixed-model association (Kang *et al.* 2008).

We have recently identified *Nox3* as an NIHL susceptibility gene in a GWAS with a subset of the strains in the HMDP (Lavinsky *et al.* 2015). Here, we describe the first completed GWAS for NIHL, and demonstrate a distinct genetic landscape for this disorder that is notably different from baseline strain variation in hearing, providing the first G × E analysis for NIHL, to date.

## Materials and Methods

### HMDP strains and genotypes

A description of the HMDP (strain selection, statistical power, and mapping resolution) is provided in (Bennett *et al.* 2010). Female mice of 100 inbred strains (3–8 animals of each strain) from the HMDP (Jackson Laboratories) were obtained and used following the University of Southern California Institutional Animal Care and Use Committee guidelines. Strains and genotype data are available from Jackson Laboratories (http://www.jax.org). Mice were 4 wk old, and to ensure adequate acclimatization to a common environment, mice were aged until 5 wk. Mice were housed in sterilized microisolator cages and received autoclaved food and water *ad libitum*.

### Baseline and postnoise exposure hearing phenotypes

For baseline and postnoise exposure auditory brainstem response (ABR) measurements, mice were anesthetized with an intraperitoneal injection of a mixture of ketamine (80 mg/kg body wt) and xylazine (16 mg/kg body wt). Stainless steel electrodes were placed subcutaneously at the vertex of the head and the right mastoid, with a ground electrode at the base of the tail. Mouse body temperature was maintained through the use of a TCAT-2DF temperature controller and the HP-4 M heating plate (Physitemp Instruments Inc., Clifton, NJ). Artificial tear ointment was applied to the eyes during anesthesia. Each mouse was recovered on a heating pad at body temperature. Stainless steel electrodes were placed subcutaneously at the vertex of the head and the left mastoid. A ground electrode was placed at the base of the tail. Test sounds were presented using an Intelligent Hearing Systems speaker attached to an 8-inch long tube that was inserted into the ear canal. Due to time and equipment constraints, only the left ear was assessed.

Auditory signals were presented as tone pips, with a rise and a fall time of 0.3 msec and a total duration of 1 msec, at the frequencies 4, 8, 12, 16, 24, and 32 kHz. Tone pips were delivered below threshold and then increased in 20-dB increments until it reached the goal of 100 dB. Intensity was then decreased in smaller steps of 10, 5, and 2 dB, as the threshold was approached. Signals were presented at a rate of 40/sec. Responses were filtered with a 0.3 to 3 kHz pass-band (×20,000). For each stimulus intensity 512 waveforms were averaged. Hearing threshold was determined by inspection of ABR waveforms and was defined as the minimum intensity at which wave 1 could be distinguished. Postexposure thresholds were evaluated by the same method, 2 wk postexposure.

### Noise exposure protocol

Mice aged 5-wk-old were exposed for 2 hr to 10 kHz octave band noise (OBN) at 108 dB sound pressure level (SPL), using a method adapted from Kujawa and Liberman (2009). The OBN noise exposure was previously described (White *et al.*, 2009). For 2 hr, mice were placed in a circular quarter-inch wire-mesh exposure cage with four shaped compartments, and were able to move about within the compartment. The cage was placed in a MAC-1 soundproof chamber designed by Industrial Acoustics (IAC, Bronx, NY) and the sound chamber was lined with soundproofing acoustical foam to minimize reflections. Noise recordings were played with a Fostex FT17H Tweeter Speaker built into the top of the sound chamber. Calibration of the damaging noise was done with a B&K sound level meter, with a variation of 1.5 dB across the cage.

A data acquisition board from National Instruments (National Instruments Corporation, Austin, TX) was regulated by custom software (used to generate the stimuli and to process the responses). Stimuli were provided by a custom acoustic system, made up of two miniature speakers, and sound pressure was measured by a condenser microphone. Testing involved the right ear only. All hearing tests were performed in a separate MAC-1 soundproof chamber to eliminate both environmental and electrical noise.

### Cochlear expression profiles in the HMDP for expression quantitative trait loci studies

Both cochleae from each 8-wk-old mouse were isolated from the 64 HMDP strains (2–4 mice/strain). The inner ear was microdissected and the surrounding soft tissue and the vestibular labyrinth was removed. The dissected cochleae were then frozen in liquid nitrogen and ground to powder. RNA was extracted and purified by placing cochlea samples in RNA lysis buffer (Ambion). The sample was incubated overnight at 4°, centrifuged (12,000 × *g* for 5 min) to pellet insoluble materials, and RNA isolated following manufacturer’s recommendations. This procedure generates ∼300 ng of total RNA per mouse.

Gene expression analysis was performed and gene expression measurements were taken using Illumina’s mouse whole genome expression kit, BeadChips. Amplifications and hybridizations were performed according to Illumina’s protocol (Southern California Genome Consortium microarray core laboratory at University of California, Los Angeles). RNA (100 ng) was reverse transcribed to cDNA using Ambion cDNA synthesis kit (AMIL1791) and then converted to cRNA and labeled with biotin. Further, 800 ng of biotinylated cRNA product was hybridized to prepare whole genome arrays and was incubated overnight (16–20 hr) at 55°. Arrays were washed and then stained with Cy3-labelled streptavadin. Excess stain was removed by washing and then arrays were dried and scanned on an Illumina BeadScan confocal laser scanner.

### Statistical analyses

GWAS analyses for hearing phenotypes in the HMDP strains were performed using genotypes of ∼500,000 single nucleotide polymorphisms (SNPs) obtained from the Mouse Diversity Array. SNPs were required to have minor allele frequencies >5% and missing genotype frequencies <10%. Applying these filtering criteria resulted in a final set of ∼200,000 SNPs that were used for analysis. Association testing was performed using FaST-LMM (Lippert *et al.* 2011) a linear mixed-model method that is fast and able to account for population structure. To improve power when testing all SNPs on a specific chromosome, the kinship matrix was constructed using the SNPs from all other chromosomes. This procedure includes the SNP being tested for association in the regression equation only once. Genome-wide significance threshold in the HMDP was determined by the family-wise error rate (FWER) as the probability of observing one or more false positives across all SNPs per phenotype. We ran 100 different sets of permutation tests and parametric bootstrapping of size 1000 and observed that the genome-wide significance threshold at a FWER of 0.05 corresponded to *P* = 4.1 × 10^−6^, similar to that used in previous studies with the HMDP (Bennett *et al.*, 2010). This is approximately an order of magnitude larger than the threshold obtained by Bonferroni correction (4.6 × 10^−7^), which would be an overly conservative estimate of significance because nearby SNPs among inbred mouse strains are highly correlated with each other.

RefSeq genes were downloaded from the UCSC genome browser (https://genome.ucsc.edu/cgi-bin/hgGateway) using the NCBI Build37 genome assembly to characterize genes located in each association. The 95% confidence interval for the distribution of distances between the most significant and the true causal SNPs, for simulated associations that explain 5% of the variance in the HMDP, is 2.6 Mb (Bennett *et al.* 2010). Only SNPs mapping to each associated region were used in this analysis. We selected SNPs that were variant in at least one of the HMDP CI strains. Nonsynonymous SNPs within each region were downloaded from the Mouse Phenome Database (http://phenome.jax.org/).

In order to evaluate the G × E interactions, we performed an association analysis that exploits the natural genetic and phenotypic variation of hearing levels provided in (Crow *et al.*, 2015). For environmental effects, we compared loci mapped at baseline *vs.* postnoise exposure in the same population.

The Pearson coefficient (*r*) was used to assess correlations between our baseline and postnoise exposure ABR thresholds, A two-tailed *P*-value <0.05 indicated statistically significant differences. The strength was described as: weak (<0.50), moderate (0.50–0.70), and strong (>0.70).

### Data availability

The authors state that all data necessary for confirming the conclusions presented in the article are represented fully within the article.

## Results

### Large frequency specific strain variation in susceptibility to NIHL in mice

**Figure 1:**
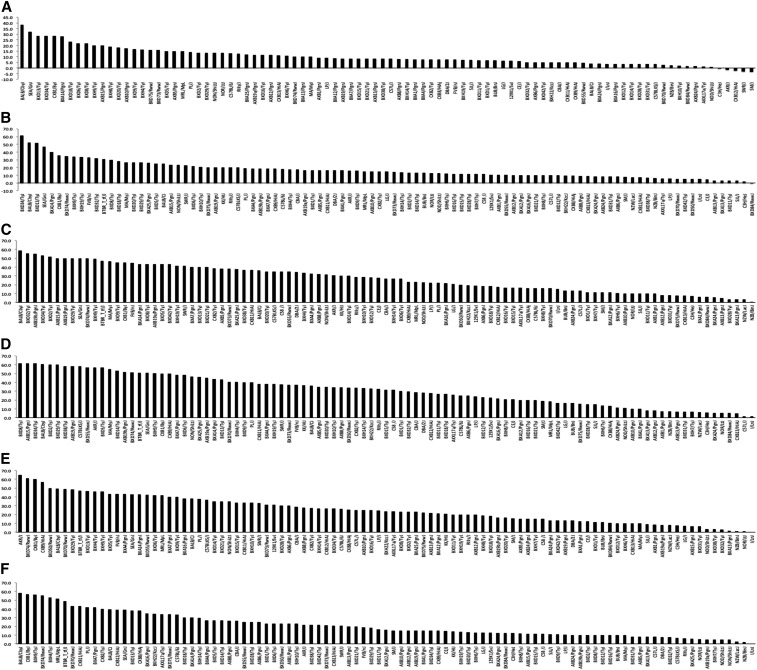
Effects of genetic background on postnoise exposure ABR thresholds at 4 kHz (A), 8 kHz (B), 12 kHz (C), 16 kHz (D), 24 kHz (E), and 32 kHz (F) tone burst in 100 HMDP inbred strains. The difference between the strains with the lowest and the highest values were 2.13- (4 kHz), 3.16- (8 kHz), 4.34- (12 kHz), 3.72- (16 kHz), 3.53- (24 kHz), and 3.74-fold (32 kHz). Several strains demonstrated variable susceptibility depending on the frequency tested. The most susceptible strains at 4, 8, 12, 16, 24, and 32 kHz were SEA/GnJ, BXD16/TyJ, NOR/LtJ, BXD32/TyJ, DBA/2J, and AXB15/PgnJ, respectively.

As an essential first step in characterizing the genetic architecture of strain variation in susceptibility to NIHL, we recorded ABR thresholds both preexposure and 2 wk after a 2-hr exposure to a 10-kHz OBN at 108 dB SPL in 5–6-wk-old female mice from the HMDP (Myint *et al.* 2016). These data demonstrated strain variation in ABR thresholds postnoise exposure, suggesting a genetic component (Supplemental Material, Figure S1). Figure 1 demonstrates the results for postnoise exposure ABR thresholds after tone burst stimuli of 4, 8, 12, 16, 24, and 32 kHz in the HMDP. Among the 100 strains characterized, ABR thresholds across the frequencies tested varied by 2.13- to 4.34-fold.

There were significant correlations between our ABR postnoise exposure phenotypes at different frequencies (Table 1). These correlations were strong (*r* > 0.7) between the neighboring frequencies, such as those between 8 and 12 kHz, 12 and 16 kHz, 16 and 24 kHz, and 24 and 32 kHz. Conversely, higher and lower frequencies were weakly correlated (*r* < 0.5). These data are consistent with those from our unexposed cohort in which we found the genetic architecture to be highly frequency specific (Crow *et al.* 2015).

**Table 1:** Correlations between ABR postnoise exposure phenotypes at different frequencies

ABR Thresholds	Correlation Test	4 kHz	8 kHz	12 kHz	16 kHz	24 kHz	32 kHz
4 kHz	Pearson correlation	1	0.800**	0.445**	0.305**	0.364**	0.327**
	Sig. (two-tailed)		0.000	0.000	0.000	0.000	0.001
8 kHz	Pearson correlation	0.800**	1	0.703**	0.552**	0.479**	0.371**
	Sig. (two-tailed)	0.000		0.000	0.000	0.000	0.000
12 kHz	Pearson correlation	0.445**	0.703**	1	0.894**	0.768**	0.622**
	Sig. (two-tailed)	0.000	0.000		0.000	0.000	0.000
16 kHz	Pearson correlation	0.305**	0.552**	0.894**	1	0.865**	0.698**
	Sig. (two-tailed)	0.003	0.000	0.000		0.000	0.000
24 kHz	Pearson correlation	0.364**	0.479**	0.768**	0.865**	1	0.864**
	Sig. (two-tailed)	0.000	0.000	0.000	0.000		0.000
32 kHz	Pearson correlation	0.327**	0.371**	0.622**	0.698**	0.864**	1
	Sig. (two-tailed)	0.001	0.000	0.000	0.000	0.000	

** Correlation significant at *P* = 0.01 (two-tailed). Sig., significance.

### Cochlear sensitivity to noise is independent of preexposure hearing ability

In an effort to understand the similarities and differences of preexposure and postexposure phenotypes, we ascertained whether baseline hearing thresholds were correlated with those after noise exposure. To accomplish this, we analyzed pairwise comparisons between the two measures for all combinations of baseline and postexposure frequencies for the 100 HMDP strains. We discovered that several strains had severe baseline hearing deficits at certain frequencies that made subsequent calculations of postexposure thresholds unreliable. This phenomenon has been termed the “ceiling effect” by Lin *et al.* (2009), and is explained by the ear having a set number of damage-susceptible elements, such that if more elements are damaged at baseline, there are fewer potential elements that can be damaged as a result of noise exposure. To mitigate this ceiling effect, we elected to use an inclusion criteria of <40 dB threshold at baseline, as this appeared to be a natural lower limit of baseline hearing impairment. This categorization comes from our observations of the HMDP strains, as presented in our recent paper describing the range of NIHL in the HMDP (Myint *et al.* 2016). As all strains showed baseline thresholds >40 dB at the 4 kHz frequency, no correlation test was performed between baseline and postexposure threshold at 4 kHz.

**Figure 2:**
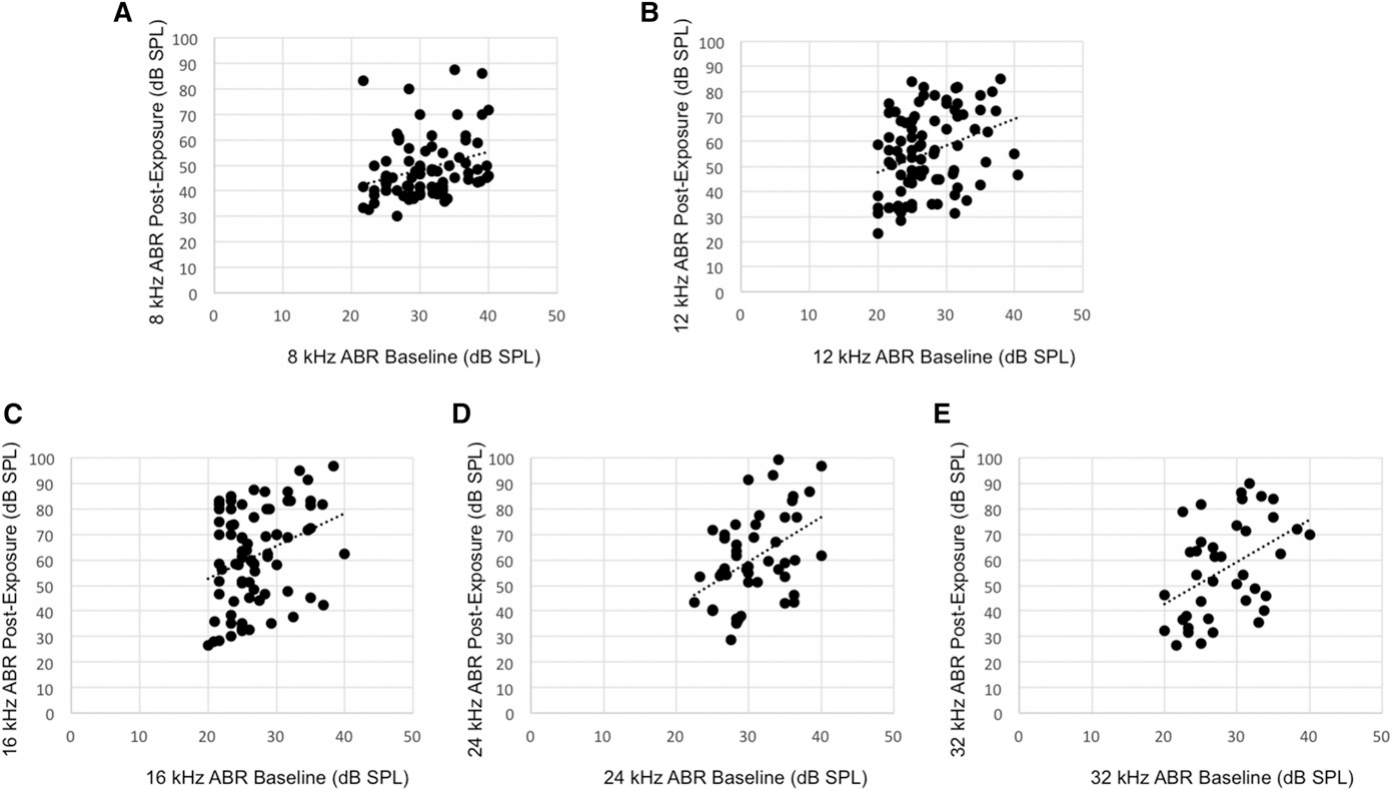
Pairwise correlations between baseline ABR threshold and postnoise exposure threshold for 8, 12, 16, 24, and 32 kHz (A, B, C, D, and E, respectively) after exclusion of baseline hearing impaired strains. These plots were selected as they were representative of the correlations delineated in Table 2.

Pairwise correlations between baseline ABR thresholds and postexposure thresholds for each of the HMDP strains revealed weak correlations (*r* < 0.5) when corrected for multiple comparisons (Figure 2 and Table 2), and this contributed to provide more evidence for G × E interactions in NIHL. These analyses suggest that although there exists strain variation in preexposure and postexposure hearing thresholds, the genetic underpinnings are likely different.

**Table 2:** Summary of Pearson *r* calculations for each pairwise correlation between baseline and postexposure ABR thresholds of the 100 HMDP strains after excluding strains with baseline thresholds >40 dB to control for ceiling effect

Postexposure Threshold	Baseline Threshold				
	8 kHz	12 kHz	16 kHz	24 kHz	32 kHz
8 kHz	−0.13	0.01	−0.09	0	0.05
12 kHz	−0.16	0.02	−0.02	−0.14	−0.17
16 kHz	0	0.15	0.08	−0.07	−0.06
24 kHz	−0.02	0.06	0.23	0.21	0.11
32 kHz	0.13	0.23	0.23	0.19	0.19

The 4 kHz frequency was completely excluded as no strains had baseline thresholds <40 dB.

### Transcriptomic analysis of the cochlea

We next used our cochlear gene expression database profile from the HMDP strains to identify the top 500 genes whose expression variation correlated with ABR hearing thresholds after noise exposure (Table S1). In the cochlea, *Ceacam16* had the highest statistical significance (*P*-value) with ABR hearing thresholds (*r* = 0.63, *P* = 2.34 × 10^−6^). The protein encoded by *Ceacam16* is a secreted glycoprotein that interacts with tectorial membrane proteins in the inner ear. The encoded adhesion protein is found in cochlear outer hair cells and appears to be important in several frequencies (Kammerer *et al.* 2012). Also, defects in *Ceacam16* are a cause of nonsyndromic autosomal dominant hearing loss (Wang *et al.* 2015).

By comparison, two other correlated genes, *Clrn1* and *Elmod1*, have been implicated in hearing. *Clrn1* (*r* = 0.51, *P* = 2.54 × 10^−4^) encodes a protein that may be important in the development and homeostasis of the inner ear. Also, mutations within this gene have been associated with Usher syndrome type IIIa (Tian *et al.* 2009). *Elmod1* (*r* = −0.505, *P* = 3.35 × 10^−4^) regulates the actin dynamics that determine stereocilia length in mammalian inner hair cells (Johnson *et al.* 2012). A genetic predisposition to perturbation of either endolymphatic homeostasis or stereociliary architecture after noise exposure could account for these correlations and warrants further investigation.

In another approach, we used the DAVID knowledge base (http://david.abcc.ncifcrf.gov/) to determine whether the top 500 cochlear expressed genes correlating with ABR hearing thresholds after noise exposure were enriched for specific gene ontology (GO) categories. The DAVID functional annotation clustering tool was used to identify significant (enrichment score (ES) > 3.0) gene clusters containing highly related GO terms. The top cluster contained genes involved in: 1) olfactory transduction/receptor (8.3-fold, adjusted *P* = 9.5 × 10^−14^), 2) sensory perception (2.9-fold, adjusted *P* = 4.3 × 10^−7^), and 3) neurological system processes (2.3-fold, adjusted *P* = 1.2 × 10^−6^) (Table S2). Interestingly, the *CDH23* gene is part of a cluster of genes involved in sensory perception. The *CDH23* gene is member of the cadherin family, whose genes encode calcium dependent cell–cell adhesion glycoproteins. The encoded protein is involved in stereocilia organization and hair bundle formation. *CDH23* is located in a region containing the human deafness loci DFNB12 and USH1D. Mice homozygous for null mutations in cadherin 23 genes are deaf and have disorganized stereocilia bundles. Furthermore, NIHL in heterozygous is two times greater than for wild-type littermates (Kowalski *et al.* 2014).

### Identification of genetic loci contributing to NIHL

**Figure 3:**
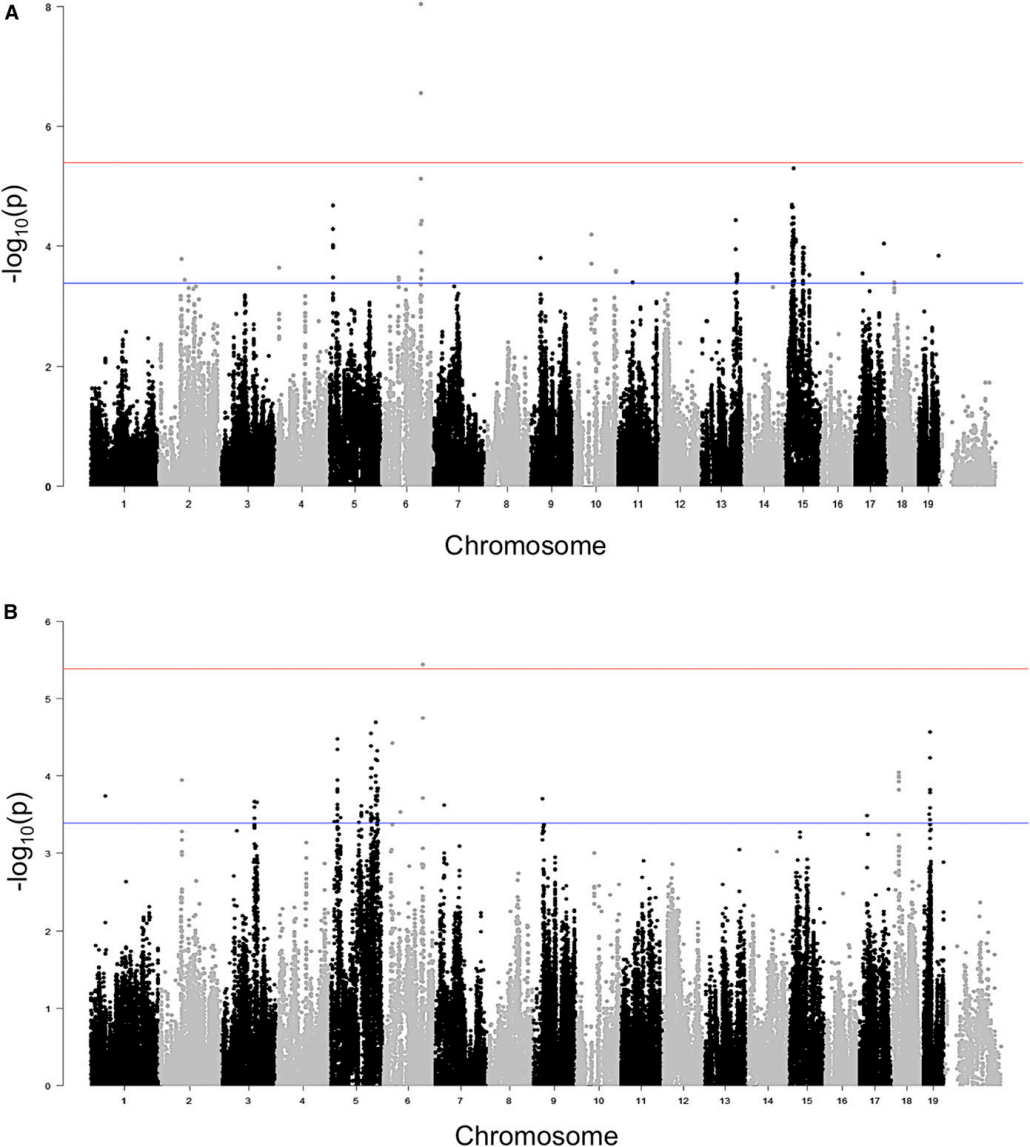
(A) GWAS results for 32 kHz postnoise exposure thresholds in the HMDP. Manhattan plot showing the association (−log10) *P*-values (−log*P*) for 32 kHz in 100 HMDP inbred mouse strains. The analysis was performed using >200,000 SNPs with a minor allele frequency >5%. Each chromosome is plotted on the *x*-axis in alternating brown and blue colors. SNP on chromosome 6 exceeded the predetermined genome-wide significance threshold (*P* = 4.1 × 10^−6^). (B) GWAS results for 24 kHz postnoise exposure thresholds in the HMDP. Manhattan plot showing the association (−log10) *P*-values (−log*P*) for 24 kHz in 100 HMDP inbred mouse strains. The analysis was performed using >200,000 SNPs with a minor allele frequency >5%. Each chromosome is plotted on the *x*-axis in alternating brown and blue colors.

We next performed a GWAS for postexposure hearing thresholds (Figure 3, A and B) to identify loci associated with NIHL at the various frequencies tested (Figure S2 and S3).

**Figure 4:**
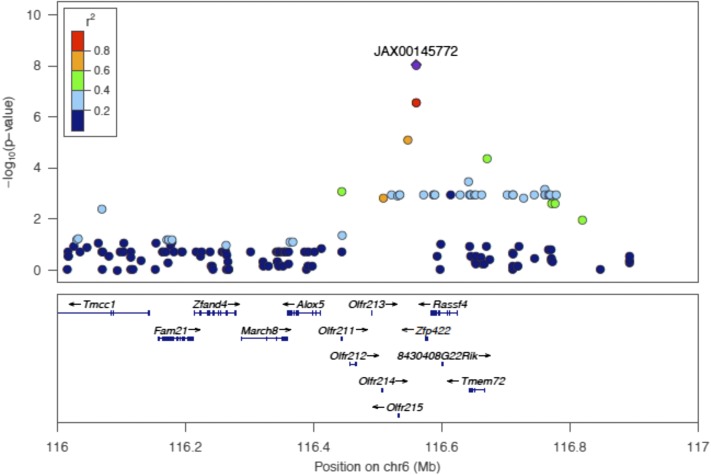
Regional plot of the 24 kHz and 32 kHz ABR threshold on chromosome 6 association in the HMDP centered on the most significant SNP (rs37517079). The blue diamond represents the most significant SNP (*P* = 9.8 × 10^−9^) at 32 kHz. Phenotype and SNPs are colored based on their linkage disequilibrium (LD) with the most significant SNP being red SNPs in LD at r2 > 0.8, orange SNPs in LD at coefficient of determination (r2) > 0.6, and green SNPs in LD at r2 > 0.4. The positions of all RefSeq genes are plotted using genome locations (NCBI’s Build37 genome assembly). The value on the *y*-axis represents the −log10 of the *P*-value and corresponds to the genome-wide significance.

Postexposure ABR threshold associations exceeding the genome-wide significance threshold were identified on chromosome 6 at the 32 kHz and 24 kHz (Figure 4 and Table 3) tone burst stimuli with the identical peak SNP (rs37517079). These data, in conjunction with our correlational analysis, support the concept that the genetic architecture of NIHL is frequency specific (Table 3). We next focused our attention on candidate genes within this novel interval.

**Table 3:** Locus identified in HMDP by GWAS for ABR phenotypes after noise exposure

Trait*^a^*	Chr	SNP*^b^*	Position (Mb)*^c^*	−log*P*	MAF	No. of Genes*^d^*
ABR 24 kHz (post)	6	rs37517079	116.6	3.66 × 10^−6^	0.120	14
ABR 32 kHz (post)	6	rs37517079	116.6	9.8 × 10^−9^*	0.120	14

* Genome-wide significant: *P* < 4.1 × 10^−6^. MAF, minor allele frequency.

a ABR postnoise exposure hearing thresholds in different frequencies.

b The most significant SNP.

c Locations based on genome assembly (NCBI´s Build37)

d Number of RefSeq genes (NCBI´s Build37 assembly) located in the mouse association interval. The 95% confidence interval for the distribution of distances between the most significant and the true causal SNPs, for simulated associations that explain 5% of the variance in the HMDP, is 2.6 Mb.

There are several strategies by which candidate genes can be selected within GWAS intervals, including known expression patterns, implication of involvement in the phenotype under study, and the use of tissue-specific expression quantitative trait loci (eQTL) data. We explored our interval for the presence of cochlear *cis* eQTLs.

### eQTL

**Figure 5:**
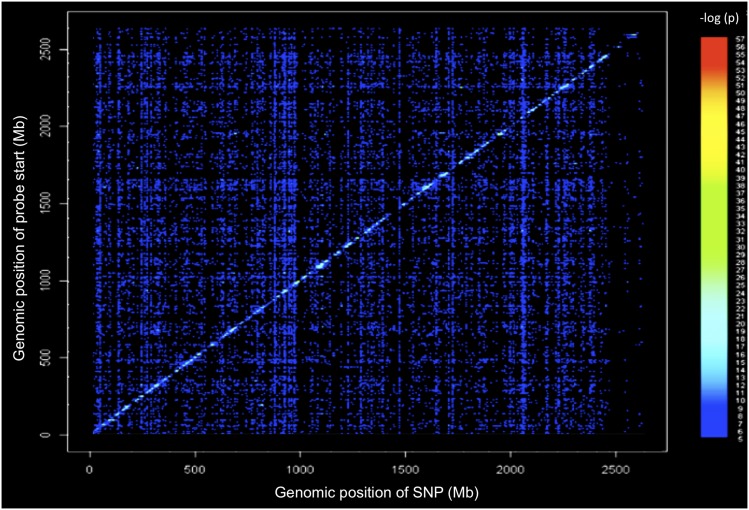
Cochlear eQTL plot. Diagonal line represents *cis* eQTLs. Dense vertical lines represent *trans*-eQTL hotspots. *x*-axis, expression SNP (eSNP) position; *y*-axis, probe position.

To model causal interactions, help prioritize candidate genes at GWAS loci, and examine gene-by-gene interactions, we mapped loci controlling microarray-generated gene expression traits (eQTL) in the cochlea of 64 HMDP strains (Figure 5). Loci in which peak SNPs mapped to within 2 Mb of the gene whose expression was regulated were considered “local” or *cis*-acting eQTL, while SNPs mapping elsewhere were considered “distal” and presumably *trans*-acting eQTL. We calculated the significant *P*-value cutoff (*P* = 1 × 10^−6^) for local and distal associations. A total of 18,138 genes were represented by at least one probe, after excluding probes that overlapped SNPs present among the CI strains used in the HMDP corresponding to 1% false discovery rate (see *Materials and Methods*).

The locus for NIHL had at least one gene within the association peak regulated by a significant local cochlear eQTL. Of the 14 candidate genes at the locus, we identified five as having significant *cis* eQTL in the cochlea (Table 4): *Rassf4*, *March8*, *Zfp422*, *Olfr212*, and *Olfr215*. *Rassf4* may play a role in tumor suppression (Eckfeld *et al.* 2004), *March8* induces the internalization of several membrane glycoproteins (Eyster *et al.* 2011), *Zfp422* may play a role in osteogenic development (Ganss *et al.* 2002), and *Olfr212* and *Olfr215* are part of the olfactory receptor gene family (Zhang and Firestein 2002).

**Table 4:** Genes within NIHL association peaks regulated by a significant local eQTL in the cochlea

Gene	RefSeq	Chr	txStart (bp)*^a^*	txEnd (bp)*^b^*	Local eQTL *P*-value*^c^*
*Rassf4*	ILMN_2956095	6	962600683	962641511	6.22 × 10^−9^
*March8*	ILMN_1242531	6	962305610	962377215	6.88 × 10^−9^
*Zfp422*	ILMN_2435673	6	962591691	962596674	6.80 × 10^−7^
*Olfr212*	ILMN_1250164	6	962474191	962485640	7.47 × 10^−7^
*Olfr215*	ILMN_2673515	6	962549613	962550706	1.87 × 10^−6^

a txStart, location of transcription (NCBI Build37 genome assembly) start.

b txEnd, location of transcription (NCBI Build37 genome assembly) end.

c Statistically significant *P*-value ≤ 5.1 × 10^−6^ (Bonferroni corrected for the number of probes tested).

We next evaluated our *trans*-eQTL “hotspots.” These hotspots regulate the levels of many transcripts and may perturb entire pathways and mediate complex gene-by-gene and G × E interactions (van Nas *et al.* 2010). The genome was divided into 2-Mb bins and the number of significant distant eQTL were counted in each bin. The eQTL hotspots were determined by the enrichment of gene expression traits that mapped to the same loci. From Figure 5, it is evident that there are loci present that affect the expression level of transcripts distant to the gene encoding the transcript (*trans*-eQTL). We analyzed each SNP in the *trans*-eQTL hotspot on chromosome 6 and none were in the region of our peak GWAS SNP. The dense vertical bands (Figure 5) represent *trans*-eQTL hotspots that denote distant loci that impact the expression of hundreds of genes. There is clearly one such hotspot on chromosome 6 that does not overlap with our mapped locus. A future in-depth analysis of these hotspots will provide additional tools for capturing the networks of genes involved in normal and perturbed hearing.

The DAVID knowledge base was used to determine if the top 300 *trans*-eQTL hotspots were enriched for specific GO categories (Table S3). DAVID’s functional annotation clustering tool was used to identify significant (ES > 3.0) gene clusters containing highly related GO terms. The top functional annotation cluster contained genes involved in mitochondrial activity (1.8-fold, adjusted *P* = 1.30 × 10^−9^). We, as well as others, have demonstrated a role for oxidative metabolism in hearing loss associated with noise exposure (Lavinsky *et al.* 2015).

### Discovering environmentally specific loci

In order to validate the G × E effects, we compared the effects of the 32 kHz postnoise exposure (rs37517079) locus with those we reported previously from the same unexposed strains (Crow *et al.* 2015). There was no overlap between the loci detected in the baseline hearing GWAS and those in the present study. For example, our peak NIHL SNP (rs37517079) was associated with noise susceptibility at 32 kHz, but not associated with baseline or preexposure hearing, as shown by the association significance level and effect size (Table 5).

**Table 5:** Effect size at baseline *vs.* postnoise exposure with peak SNP on chromosome 6

SNP*^a^*	−log*P**^b^* Preexposure	−log*P**^c^* Postexposure	SNP Weight*^d^* (dB) Preexposure	SNP Weight*^e^* (dB) Postexposure
rs37517079	1.15 × 10^−4^	9.8 × 10^−9^*	−0.422	0.605

* Genome-wide significant: *P* < 4.1 × 10^−6^.

a The most significant SNP.

b Association (−log10) *P*-values (−log*P*) for ABR 32 kHz at baseline in 100 HMDP inbred mouse strains.

c Association (−log10) *P*-values (−log*P*) for ABR 32 kHz after exposure to noise in 100 HMDP inbred mouse strains.

d Environmental effect size (dB) for ABR 32 kHz at baseline.

e Environmental effect size (dB) for ABR 32 kHz after exposure to noise.

## Discussion

In the United States, nearly 10% of the total population is exposed to hazardous levels of daily noise in the workplace (Dobie 2008). The most extreme work place environment for NIHL is the Armed Forces. According to the Department of Veterans Affairs (VA), hearing loss is the most common disability among U.S. troops in the Middle East. The financial impact of these disability claims on the VA is staggering and likely will continue to grow. Risk could be reduced with a better understanding of the biological processes that modulate susceptibility to damaging noise.

There is a clear heritable component to NIHL in humans. Estimates from human twin studies suggest that the heritability of NIHL is ∼36% (Heinonen-Guzejev *et al.* 2005). Candidate gene studies using SNPs have identified a small number of potential susceptibility genes (Van Eyken *et al.* 2007; Fortunato *et al.* 2004; Van Laer *et al.* 2006; Konings *et al.* 2007; Konings *et al.* 2009). Unfortunately, many of these studies have low statistical power due to small sample sizes and lack of replication. Finally, the combined risk from these alleles fails to account for the majority of the genetic risk. These observations served as motivating factors for studying the genetics of this complex trait in the mouse.

Identifying G × E interactions in risk prediction models may have important implications for public health and will become an increasingly significant component in healthcare in the age of personalized medicine (Thomas 2010b). There are major challenges to the study of these interactions in humans, including exposure assessment, sample size, and heterogeneity (Thomas 2010a). It is rare that researchers are able to obtain accurate exposure measurements over a lifetime, thus limiting phenotypic accuracy. This is a major barrier to the study of NIHL in humans. Furthermore, adequately powered sample sizes for G × E studies will require thousands of subjects, which is at least four times the sample size for detecting main effects of comparable size (Smith and Day 1984). Buoyed by the successes of human GWASs and the difficulties inherent in G × E studies in humans, many investigators have turned to similar approaches in mice. There are several advantages that we used in our HMDP study, including our ability to control environmental exposure (noise), access to cochlear tissues for gene expression analyses, and the availability of a renewable set of inbred mouse strains for study.

For almost 50 years the mouse has been an essential animal model for studies in hearing loss. Different susceptibilities to NIHL have been seen in different inbred stains of mice both in our laboratory (White *et al.* 2009) and others (Johnson *et al.* 2012; Davis *et al.* 2001; Li 1992). This indicates that a significant component of the hearing loss associated with noise exposure is heritable. Several strain-specific loci for age-related hearing loss are also associated with NIHL susceptibility (Erway *et al.* 1996).

The data from the mouse thus far have demonstrated strain-dependent variability in the degree of noise damage, the time course over which damage occurs, and the impact on cochlear cell types. This body of work has clearly shown that inbred strains of mice display significant variation in their ability to absorb punishing noise (Li 1992; Erway *et al.* 1996). A number of Mendelian loci associated with NIHL have been described but the most significant locus to date is *Ahl1*, which contains the *Cdh23* gene. Although it is clear that the *Cdh23* 753A allele increases susceptibility to NIHL, results from a few studies, including our own, demonstrate that additional loci in the genomes of inbred mice contribute to this susceptibility (Vázquez *et al.* 2004; White *et al.* 2009; Li 1992; Harding *et al.* 2005). The functional analysis of natural variation holds great promise for elucidating biological processes. Selection of samples from a pool of mutations that have occurred over time, such as those found in a population, will reveal natural variants with different properties compared to experimentally induced mutations. Here, we describe the first GWAS for NIHL and demonstrate, for the first time, a clear G × E interaction for this trait.

We have recently characterized 100 strains at baseline and after noise exposure using ABR thresholds and permanent thresholds shifts. We identified several distinct patterns of noise sensitivity. This demonstration of strain variation in noise sensitivity led us to conduct this GWAS and facilitated our high-resolution mapping. Also, these data provided a complete phenotypic dataset available for general use (Myint *et al.* 2016).

We performed a GWAS for postexposure hearing thresholds and permanent threshold shift to identify loci associated with NIHL at the various frequencies tested. However, we demonstrated associations exceeding the genome-wide significance using postexposure thresholds only. There are several reasons for these findings. Several strains had severe baseline hearing deficits and made subsequent calculations of permanent threshold shift unreliable (the ceiling effect). Thus, some strains have fewer potential elements that can be damaged as a result of noise exposure.

Consistent with the clinical observation in humans that NIHL, and most ototoxins, affect the high frequency function of the cochlea, our locus also mapped at the higher frequencies of the spectrum (24 kHz and 32 kHz). By integrating transcriptomic information, we demonstrated five genes within the presumed haplotype block (*Rassf4*, *March8*, *Zfp422*, *Olfr212*, and *Olfr215*) that also had significant *cis* eQTLs in the cochlea and warrant more in-depth analyses. Interestingly, none of these genes have been linked to hearing thus far. *Zfp422* participates in osteogenesis during development (Ganss *et al.* 2002) and could play a role in the development of the bony labyrinth (otic capsule). *Olfr212* and *Olfr215* are known as olfactory receptor genes (Zhang and Firestein 2002) and may also be present in other sensory organs, such as the inner ear.

Based on our cochlear gene expression database of the top 500 most correlated genes with ABR hearing thresholds after noise exposure, *Ceacam16* had the highest statistical significance. Certainly, there is a promising role of *Ceacam16* in NIHL, since it is responsible for maintaining the integrity of the tectorial membrane. Although little is known about the impact of noise exposure on the tectorial membrane, this warrants further investigation. Furthermore, our enrichment for sensory receptor and mitochondrial function suggests a set of genes and pathways in addition to our candidate genes that are worthy of investigating further in future studies.

In our previous study (Lavinsky *et al.* 2015) we identified five genome-wide significant loci with 64 strains that do not appear in our full GWAS described here. This phenomenon likely results from the addition of 36 strains, some of which do not segregate the causal and/or “tag” SNP, thereby leading to these loci no longer reaching genome-wide significance. This likely represents the addition of strains that failed to segregate the NIHL sensitivity allele. Moreover, rs37517079 was not considered genome-wide significant in our previous scanning at 24 kHz and 32 kHz. Probably, our incomplete panel (64 strains) was not powerful enough to highlight this SNP.

The findings from this and our previous studies confirm three essential concepts that have, to date, not been reported. First, rs37517079 was associated with postnoise exposure ABR thresholds at 32 kHz and 24 kHz, which, similar to our GWAS of baseline hearing, suggests the existence of frequency-specific genetic susceptibility to NIHL. Second, there do not appear to be any loci that are associated with frequencies at the lower spectrum of hearing after noise exposure, at least based on the 100 HMDP characterized in this study. This later finding is consistent with the literature on NIHL in that this is a high frequency disease. However, we cannot exclude the possibility that using other resources for mapping that might include wild strains or other reference mouse panels (*i.e.*, Collaborative Cross) could improve genomic coverage and generate additional loci at all frequencies. Lastly, these findings also demonstrate that the genetic architecture of NIHL is distinct from that of baseline hearing thresholds in G × E interactions in NIHL, using a GWAS.

### Conclusions

We report the first GWAS controlling for population structure for NIHL, with a complete set of 100 strains from the HMDP. We present several candidate genes for NIHL, several pathways that are novel and some that are implicated in this process. Most importantly, this combination and integration of the NIHL phenotype, GWAS, and cochlear transcript levels strongly suggests that G × E interactions play a major role in susceptibility to NIHL.

## 

## Supplementary Material

Supplemental Material
