# Supplementary material for: The Genetic Architecture of Noise-Induced Hearing Loss: Evidence for a Gene-by-Environment Interaction
Source: G3 (Bethesda). 2016 Aug 11;6(10):3219–28. doi: 10.1534/g3.116.032516 (PMC5068943; doi:10.1534/g3.116.032516)
Supplement: Supplemental Material [file supp_6_10_3219__index.html]

The Genetic Architecture of Noise-Induced Hearing Loss: Evidence for a Gene-by-Environment Interaction — Supplemental Material 

# The Genetic Architecture of Noise-Induced Hearing Loss: Evidence for a Gene-by-Environment Interaction

## Supplemental Material for Lavinsky *et al.*, 2016

**Files in this Data Supplement:**

- Figure S1 - ABR permanent threshold shift at 4kHz (a), 8 kHz (b), 12 kHz (c), 16 kHz (d), 24 kHz (e) and 32 kHz (f) tone burst in 100 HMDP inbred strains. (.pdf, 424 KB0
- Figure S2 - GWAS results for ABR permanent threshold shifts in the HMDP at 4kHz (a), 8 kHz (b), 12 kHz (c) and 16 kHz (d) tone burst in 100 HMDP inbred strains. (.pdf, 166 KB)
- Figure S3 - GWAS results for ABR post-noise exposure thresholds in the HMDP at 4kHz (a), 8 kHz (b), 12 kHz (c), 16 kHz (d) and 24 kHz (e). tone burst in 100 HMDP inbred strains. (.pdf, 244 KB)
- Table S1 - Top 50 cochlear genes correlated with ABR hearing thresholds after noise exposure. (.pdf, 234 KB)
- Table S2 - Top functional annotation cluster for the genes correlated with hearing after noise exposure (Enrichment Score: 6.51). (.pdf, 20 KB)
- Table S3 - Top functional annotation clusters for the 1,363 genes regulated by the top 300 *trans*-eQTL hotspots. (.pd, 122 KB)
