# Supplementary material for: The Genetic Architecture of Noise-Induced Hearing Loss: Evidence for a Gene-by-Environment Interaction
Source: G3 (Bethesda). 2016 Aug 11;6(10):3219–28. doi: 10.1534/g3.116.032516 (PMC5068943; doi:10.1534/g3.116.032516)
Supplement: Supplemental Material [file supp_g3.116.032516_FigureS1.pdf]

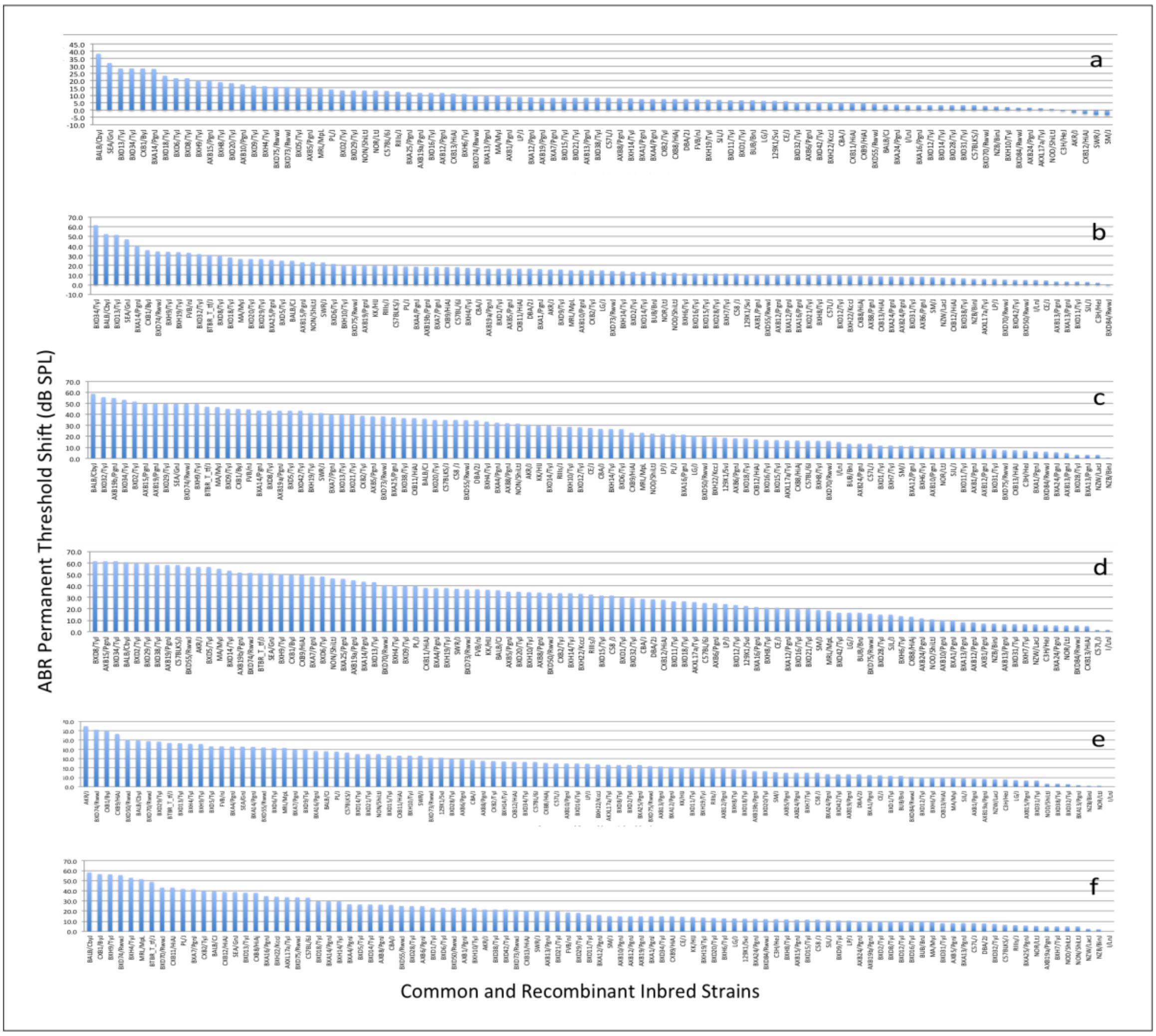

Figure S1. ABR permanent threshold shift at 4kHz (a), 8 kHz (b), 12 kHz (c), 16 kHz (d), 24 kHz (e) and 32 kHz (f) tone burst in 100 HMDP inbred strains.
