# Supplementary material for: The Genetic Architecture of Noise-Induced Hearing Loss: Evidence for a Gene-by-Environment Interaction
Source: G3 (Bethesda). 2016 Aug 11;6(10):3219–28. doi: 10.1534/g3.116.032516 (PMC5068943; doi:10.1534/g3.116.032516)
Supplement: Supplemental Material [file supp_g3.116.032516_FigureS3.pdf]

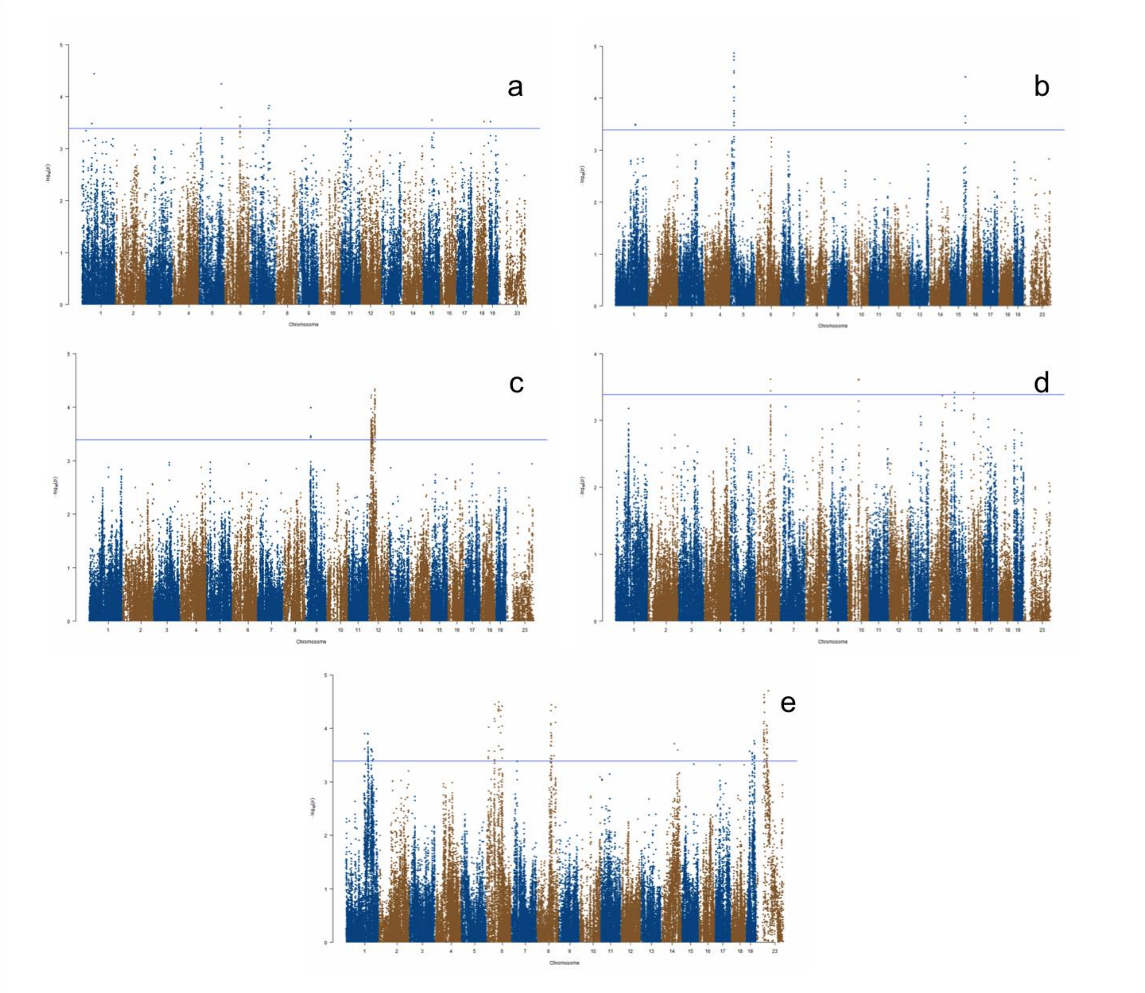

Figure S3. GWAS results for ABR post-noise exposure thresholds in the HMDP at 4kHz (a), 8 kHz (b), 12 kHz (c), 16 kHz (d) and 24 kHz (e). tone burst in 100 HMDP inbred strains. Manhattan plot showing the association (-log10) p-values (-logP) in 100 HMDP inbred mouse strains. The analysis was performed using over 200,000 SNPs with a minor allele frequency > 5%. Each chromosome is plotted on the x-axis in alternating brown and blue colors.
