# Supplementary material for: The Genetic Architecture of Noise-Induced Hearing Loss: Evidence for a Gene-by-Environment Interaction
Source: G3 (Bethesda). 2016 Aug 11;6(10):3219–28. doi: 10.1534/g3.116.032516 (PMC5068943; doi:10.1534/g3.116.032516)
Supplement: Supplemental Material [file supp_g3.116.032516_TableS1.pdf]

Table S1. Top 50 cochlear genes correlated with ABR hearing thresholds after noise exposure.

Rank	Gene Symbol	Correlation (R)	P value
**1**	*Ceacam16*	0.63314363	2.34E-06
**2**	*Nfs1*	-0.606689066	7.84E-06
**3**	*Enpp6*	0.601075866	1.00E-05
**4**	*Tpr*	0.608379381	1.18E-05
**5**	*Mageb3*	-0.581123217	2.29E-05
**6**	*Dock11*	0.580664094	2.33E-05
**7**	*Rb1*	0.580468193	2.35E-05
**8**	*Dnase1*	-0.576202531	2.78E-05
**9**	*Zfp711*	-0.57432478	3.00E-05
**10**	*Vrk2*	-0.574223342	3.01E-05
**11**	*Tyk2*	-0.573094643	3.15E-05
**12**	*Anapc10*	-0.569789802	3.58E-05
**13**	*Aldh1a7*	0.569568567	3.61E-05
**14**	*Nr5a2*	0.564941894	4.31E-05
**15**	*E130012A19Rik*	-0.56392403	4.48E-05
**16**	*Rad51l3*	-0.560016846	5.19E-05
**17**	*Npas3*	-0.558473078	5.50E-05
**18**	*2010011I20Rik*	0.557262619	5.75E-05
**19**	*Sult1d1*	0.555832289	6.07E-05
**20**	*Abca6*	0.577906441	6.13E-05
**21**	*D14Ertd500e*	-0.555050103	6.24E-05
**22**	*Prf1*	0.551855207	7.02E-05
**23**	*Tm2d3*	0.550464614	7.39E-05
**24**	*Atp7a*	0.549495335	7.65E-05

**25**	*Dnase1*	-0.548605529	7.90E-05
**26**	*Ptprc*	0.547742066	8.15E-05
**27**	*Sirpb1*	0.547181265	8.32E-05
**28**	*Ltbp4*	-0.544590872	9.13E-05
**29**	*A830006N08Rik*	0.544562809	9.13E-05
**30**	*Anapc10*	-0.540561991	0.000105271
**31**	*2010011I20Rik*	0.540231267	0.000106504
**32**	*Steap2*	-0.539824993	0.000108037
**33**	*Sirpb1*	0.538938237	0.000111453
**34**	*6430550H21Rik*	-0.538760647	0.000112148
**35**	*Txnl2*	-0.560551502	0.000112983
**36**	*Smek1*	0.536736012	0.000120364
**37**	*Ptprg*	-0.536699938	0.000120515
**38**	*Ly86*	0.536049688	0.000123271
**39**	*Samd14*	-0.535227006	0.00012684
**40**	*Ccr3*	-0.534355832	0.000130721
**41**	*Abl1*	-0.533296993	0.000135583
**42**	*Chrna2*	0.533049843	0.000136742
**43**	*Prpf38a*	0.532729091	0.000138258
**44**	*Cnfn*	0.532459001	0.000139547
**45**	*Lyz2*	-0.532216621	0.000140713
**46**	*4930522H14Rik*	0.531367649	0.000144868
**47**	*Slc6a1*	-0.530914922	0.000147128
**48**	*Klhl7*	0.529266988	0.000155631
**49**	*Olfr652*	0.529229655	0.000155828
**50**	*Abl1*	-0.528913993	0.000157509
