# Supplementary material for: The Genetic Architecture of Noise-Induced Hearing Loss: Evidence for a Gene-by-Environment Interaction
Source: G3 (Bethesda). 2016 Aug 11;6(10):3219–28. doi: 10.1534/g3.116.032516 (PMC5068943; doi:10.1534/g3.116.032516)
Supplement: Supplemental Material [file supp_g3.116.032516_TableS2.pdf]

Table S2. Top functional annotation cluster for the genes correlated with hearing after noise exposure (Enrichment Score: 6.51).

Original Database	Enriched terms	Count	P-value	Fold change	Bonferroni
KEGG_PATHWAY	**Olfactory transduction**	16	8.8E-14	8.4E0	1.1E-11
GOTERM_MF_FAT	**Olfactory receptor activity**	18	3.8E-12	5.8E0	1.9E-9
INTERPRO	**Olfactory receptor**	18	7.4E-12	5.6E0	5.4E-9
GOTERM_BP_FAT	**Sensory perception of chemical stimulus**	18	9.5e-12	5.5E0	1.7E-8
GOTERM_BP_FAT	**Sensory perception of smell**	18	9.5E-12	5.5E0	1.7E-8
PIR_SUPERFAMILY	**PIRSF003152:G protein-coupled olfactory receptor, class II**	13	1.3E-8	5.4E0	2.8E-6
GOTERM_BP_FAT	**Sensory perception**	24	4.2E-7	2.9E0	7.3E-4
GOTERM_BP_FAT	**Neurological system process**	36	3.0E-6	2.2E0	5.1E-3
GOTERM_BP_FAT	**Cognition**	28	6.9E-6	2.4E0	1.2E-2
INTERPRO	**GPCR, rhodopsin-like superfamily**	27	1.3E-5	2.4E0	9.4E-3
INTERPRO	**7TM GPCR, rhodopsin-like**	26	5.6E-5	2.2E0	4.0E-2
GOTERM_BP_FAT	**G-protein coupled receptor protein signaling pathway**	29	2.0E-4	2.0E0	2.9E-1
PIR_SUPERFAMILY	**PIRSF800006:rhodopsin-like G protein-coupled receptors**	21	2.1E-4	2.3E0	4.7E-2
GOTERM_BP_FAT	**cell surface receptor linked signal transduction**	42	5.8E-4	1.6E0	6.4E-1
SP_PIR_KEYWORDS	**G-protein coupled receptor**	24	9.3E-4	2.0E0	2.5E-1
SP_PIR_KEYWORDS	**Transducer**	26	1.9E-3	1.8E0	4.4E-1
SP_PIR_KEYWORDS	**Receptor**	47	3.3E-3	1.5E0	6.5E-1
