# Supplementary material for: The Genetic Architecture of Noise-Induced Hearing Loss: Evidence for a Gene-by-Environment Interaction
Source: G3 (Bethesda). 2016 Aug 11;6(10):3219–28. doi: 10.1534/g3.116.032516 (PMC5068943; doi:10.1534/g3.116.032516)
Supplement: Supplemental Material [file supp_g3.116.032516_TableS3.pdf]

Table S3. Top functional annotation clusters for the 1,363 genes regulated by the top 300 *trans*-eQTL hotspots.

Original database	Enriched terms	Count	P_Value	Fold Change	Bonferroni
**Annotation Cluster 1 Enrichment Score: 6.95**					
SP_PIR_KEYWORDS	mitochondrion	105	1.30E-09	1.80	5.90E-07
GOTERM_CC_FAT	mitochondrion	156	1.60E-09	1.60	7.50E-07
SP_PIR_KEYWORDS	transit peptide	64	5.20E-07	1.90	2.40E-04
GOTERM_CC_FAT	mitochondrial part	69	3.10E-06	1.80	1.50E-03
UP_SEQ_FEATURE	transit peptide:Mitochondrion	63	5.40E-06	1.80	1.50E-02
**Annotation Cluster 2 Enrichment Score: 4.12**					
GOTERM_CC_FAT	mitochondrial part	69	3.10E-06	1.80	1.50E-03
GOTERM_CC_FAT	organelle envelope	68	1.70E-05	1.70	8.10E-03
GOTERM_CC_FAT	envelope	68	1.90E-05	1.70	9.20E-03
GOTERM_CC_FAT	mitochondrial membrane	48	1.60E-04	1.80	7.30E-02
GOTERM_CC_FAT	organelle inner membrane	42	2.20E-04	1.80	1.00E-01
GOTERM_CC_FAT	mitochondrial inner membrane	40	2.90E-04	1.80	1.30E-01
GOTERM_CC_FAT	mitochondrial envelope	49	3.40E-04	1.70	1.50E-01
GOTERM_CC_FAT	organelle membrane	87	3.40E-04	1.50	1.50E-01
**Annotation Cluster 3 Enrichment Score: 3.64**					
GOTERM_CC_FAT	mitochondrial part	69	3.10E-06	1.80	1.50E-03
GOTERM_CC_FAT	mitochondrial lumen	24	2.00E-03	2.00	6.20E-01
GOTERM_CC_FAT	mitochondrial matrix	24	2.00E-03	2.00	6.20E-01
**Annotation Cluster 4 Enrichment Score: 3.28**					
GOTERM_BP_FAT	intracellular transport	58	6.60E-06	1.90	1.90E-02

SP_PIR_KEYWORDS	protein transport	60	1.30E-05	1.80	5.70E-03
GOTERM_BP_FAT	protein localization	81	3.20E-04	1.50	6.00E-01
GOTERM_BP_FAT	establishment of protein localization	72	4.00E-04	1.50	6.90E-01
GOTERM_BP_FAT	protein transport	71	5.30E-04	1.50	7.80E-01
SP_PIR_KEYWORDS	transport	145	1.50E-03	1.30	4.90E-01
GOTERM_BP_FAT	cellular protein localization	35	6.00E-03	1.60	1.00E+00
GOTERM_BP_FAT	cellular macromolecule localization	35	6.60E-03	1.60	1.00E+00
GOTERM_BP_FAT	intracellular protein transport	32	1.00E-02	1.60	1.00E+00
